# Association between walking pace and incident type 2 diabetes by adiposity level: A prospective cohort study from the UK Biobank

**DOI:** 10.1111/dom.15053

**Published:** 2023-04-03

**Authors:** Jirapitcha Boonpor, Solange Parra‐Soto, Jasunella Gore, Atefeh Talebi, Nathan Lynskey, Andrea Raisi, Paul Welsh, Naveed Sattar, Jill P. Pell, Jason M. R. Gill, Stuart R. Gray, Frederick K. Ho, Carlos A. Celis‐Morales

**Affiliations:** ^1^ School of Cardiovascular and Metabolic Health University of Glasgow Glasgow UK; ^2^ Faculty of Public Health Kasetsart University, Chalermphrakiat Sakon Nakhon Province Campus Sakon Nakhon Thailand; ^3^ Department of Nutrition and Public Health Universidad del Bío‐Bío Chillan Chile; ^4^ Center for Exercise Science and Sport, Department of Neuroscience and Rehabilitation University of Ferrara Ferrara Italy; ^5^ School of Health and Wellbeing University of Glasgow Glasgow UK; ^6^ Research Group on Education, Physical Activity and Health (GEEAFyS) University Católica del Maule Talca Chile

**Keywords:** body fat, gait, mediation, obesity, type 2 diabetes mellitus

## Abstract

**Aims:**

To investigate the combined association of adiposity and walking pace with incident type 2 diabetes.

**Methods:**

We undertook a prospective cohort study in 194 304 White‐European participants (mean age 56.5 years, 55.9% women). Participants' walking pace was self‐reported as brisk, average or slow. Adiposity measures included body mass index (BMI), waist circumference (WC) and body fat percentage (BF%). Associations were investigated using Cox proportional hazard models, with a 2‐year landmark analysis. A four‐way decomposition analysis was used for mediation and additive interaction.

**Results:**

The median (interquartile range) follow‐up was 5.4 (4.8‐6.3) years. During the follow‐up period, 4564 participants developed type 2 diabetes. Compared to brisk‐walking participants with normal BMI, those with obesity who walked briskly were at an approximately 10‐ to 12‐fold higher risk of type 2 diabetes (hazard ratio [HR] 9.64, 95% confidence interval [CI] 7.24‐12.84, in women; HR 11.91, 95% CI 8.80‐16.12, in men), whereas those with obesity and walked slowly had an approximately 12‐ to 15‐fold higher risk (HR 12.68, 95% CI 9.62‐16.71, in women; HR 15.41, 95% CI 11.27‐21.06, in men). There was evidence of an additive interaction between WC and BF% and walking pace among women, explaining 17.8% and 47.9% excess risk respectively. Obesity mediated the association in women and men, accounting for 60.1% and 44.9%, respectively.

**Conclusions:**

Slow walking pace is a risk factor for type 2 diabetes independent of adiposity. Promoting brisk walking as well as weight management might be an effective type 2 diabetes prevention strategy given their synergistic effects.

## 1 INTRODUCTION

Type 2 diabetes is a global public health challenge, given that 9.3% of individuals aged 20 to 79 years have known type 2 diabetes worldwide,^1^ and global projections estimate that type 2 diabetes prevalence will increase to 10.9% by 2045.^2^ Obesity is one of the most important modifiable risk factors for type 2 diabetes.^3^, ^4^ Since obesity is a relatively stable phenotype with limited effective interventions,^5^ it is important to identify factors that might protect people with obesity from developing type 2 diabetes.

Previous studies have reported that the association of obesity with diabetes risk^6^, ^7^, ^8^ varies by physical activity level, indicating that people with obesity should consider adopting a higher level of physical activity to manage weight as well as to reduce type 2 diabetes risk.^8^ A recent study that included 117 878 participants from nine prospective studies reported that, compared to normal‐weight but highly physically active individuals, those with obesity and had low physical activity levels had a 7.4 times higher risk of type 2 diabetes.^8^ However, most of the evidence is restricted to total physical activity, with limited evidence on other common forms of physical activity, such as walking pace.^8^ Usual walking pace has been identified as a strong predictor of poorer health outcomes.^9^ A recent study has suggested that walking pace could even improve predictive ability in identifying individuals at high risk of cardiovascular disease incidence, beyond that of traditional risk factors.^10^ Despite this, current evidence mainly focuses on stratified analysis or inclusion of adiposity as a covariate in statistical analysis.^11^ No evidence is available on the potential additive association between walking pace combined with obesity and type 2 diabetes risk, or whether such an association differs according to different adiposity markers. Moreover, how much of the association between walking pace and type 2 diabetes is mediated by adiposity has not been addressed.

Understanding the interplay between adiposity and walking pace in type 2 diabetes risk could inform future interventions aiming to increase physical activity or reduce adiposity for type 2 diabetes prevention. The aim of this study, therefore, was to investigate the combined association of adiposity and walking pace with incident type 2 diabetes in a large prospective cohort study and to understand the extent to which the higher risk of type 2 diabetes associated with slow walking may be mediated by adiposity.

## 2 MATERIALS AND METHODS

### 2.1 Study population

The UK Biobank recruited >502 000 participants from the general population between 2006 and 2010 (5.5% response rate, men and women aged 37‐73 years). Participants attended one of 22 assessment centres across England, Wales and Scotland. At the baseline assessment, an electronically signed consent form and a touchscreen questionnaire were completed. Physical measurements and biological samples were also collected, including blood, urine and saliva.^12^, ^13^ Analyses for the current study were conducted in 194 304 White‐European participants from the UK Biobank cohort (age 38‐71 years), who had available data records from primary care, exposures, and covariates. Participants with prevalent diabetes (type 1 or type 2 diabetes; n = 12 967) or undiagnosed diabetes (HbA1c ≥ 48 mmol/mol; n = 1589) at baseline were excluded from the analysis. Additionally, participants from non‐White ethnic backgrounds (n = 8390) or who had missing data (n = 11 199) were excluded from the study (Figure S1).

### 2.2 Adiposity definitions

Anthropometric measurements were measured by trained research nurses using standardized protocols.^14^ All anthropometric measurements were taken barefoot and with light clothing. Body composition in this study were measured in a fasting state. Briefly, participants were asked not to eat or drink (except plain water) for up to 4 hours before they visited the assessment centres. They were asked to go to the physical measurement station before going to the sample collection station.^14^ Body weight and body fat percentage (BF%) were assessed using a Tanita BC418MA body composition analyser. Height was measured, without shoes, using the wall‐mounted SECA 240 height measure. Waist circumference (WC) was measured midway between the lowest rib margin and the iliac crest, horizontally, using a non‐elastic SECA 200 tape measure. For WC and BF%, sex‐specific tertiles were derived and categorized as low, middle or high (46‐92 cm, 78.1‐101 cm and 89.1‐182 cm for WC; 5%‐33.8%, 23.1%‐39.8% and 27.9%‐69.3% for BF%). BMI was calculated from weight (kg) divided by the square of height (m). BMI was classified as normal weight (18.5‐24.9 kg/m^2^), overweight (25‐29.9 kg/m^2^) or obesity (≥30 kg/m^2^). Underweight was not included in the present analysis. Centrally obese WC was defined as WC ≥88 cm for women and ≥102 cm for men. High BF% was defined as >35% for women and >25% for men.^15^

### 2.3 Walking pace assessment

Self‐reported usual walking pace was collected at baseline through a touchscreen questionnaire. Participants were asked, “How would you describe your usual walking pace?”. Participants could select any of the three following answers: slow pace (defined as less than 3 miles per hour), steady average pace (defined as 3‐4 miles per hour) and brisk pace (defined as more than 4 miles per hour), as described elsewhere^16^; thus, walking pace was categorized by each participant as slow, average or brisk walking pace.

### 2.4 Ascertainment of type 2 diabetes

Incident type 2 diabetes was derived from linkage to primary care data in the UK Biobank. Records were extracted for 45% of the UK Biobank cohort. The end of coverage (extract date) was May 2017 for Scotland, September 2017 for Wales and August 2017 for England. Detailed linkage procedures are available at http://biobank.ndph.ox.ac.uk/showcase/showcase/docs/primary_care_data.pdf. We defined incident type 2 diabetes as diagnosis in primary care with International Classification of Diseases, 10th revision (ICD‐10) code E11. READ codes used in the primary care data were converted into ICD‐10 codes using the UK Biobank look‐up table. Undiagnosed type 2 diabetes was defined as glycated haemoglobin (HbA1c) values in the diabetic range (≥ 48 mmol/mol), as per the American Diabetes Association guidelines for type 2 diabetes diagnosis (https://diabetes.org/diabetes/a1c/diagnosis).

### 2.5 Covariates

Sociodemographic variables included: sex; education level (College or University degree, A levels/AS levels or equivalent, O levels/GCSEs or equivalent, CSEs or equivalent/NVQ or HND or HNC), self‐reported at baseline; age, calculated from the date of birth and date of baseline assessment; and deprivation index, an area‐based measure of socioeconomic status, derived from the postal code of residence using the Townsend deprivation score.^17^

Lifestyle variables included: smoking status (never, former, current); fruit and vegetable intake (portions per day); red meat intake (portions per week); processed meat intake (times per week); alcohol consumption (daily/almost daily, 3‐4 times a week, once or twice a week, 1‐3 times a month, special occasions, never); and sleep duration (hours per day), and were determined via self‐report questionnaire. Total sedentary time was also self‐reported and was derived from the sum of TV viewing time, computer screen time and time spent driving during leisure time and reported as hours per day.

Multimorbidity was derived from participants who self‐reported having chronic illnesses at baseline, based on 43 long‐term conditions, and was categorized as the number of existing morbidities. Medication for cardiometabolic diseases (cardiovascular medications, thiazides, steroids and statins) was also self‐reported by the participants at the baseline assessment. Additional details about these measurements can be found in the UK Biobank online protocol (https://www.ukbiobank.ac.uk/).

### 2.6 Statistical analysis

Baseline characteristics of the study population were described as mean and standard deviation (SD) for continuous variables, and frequency and percentage (%) for categorical variables. Cox proportional hazard models were used to investigate the independent association of walking pace and adiposity levels (based on BMI, WC and BF%) with incident type 2 diabetes, with years of follow‐up used as timeline variables. The associations were reported as hazard ratios (HRs) and their 95% confidence intervals (CIs). The associations between adiposity level and walking pace and incident type 2 diabetes were investigated using nine categories derived from combinations of the adiposity variables (low, middle, high, normal weight, overweight and obesity) and walking pace (brisk, average and slow pace). The analyses were conducted using a 2‐year landmark period, which excluded any incident type 2 diabetes occurring in the first 2 years of the follow‐up, to minimize reverse causation.

The analyses of the individual associations between walking pace and adiposity with incident type 2 diabetes were adjusted in an incremental manner for covariates using two models and were conducted separately for women and men. Model 1 was adjusted for sociodemographic and lifestyle factors, including age, deprivation index, education, smoking status, fruit and vegetable intake, red meat intake, processed meat intake, alcohol intake, total sedentary time, sleep duration and multimorbidity. Model 2 was adjusted as for Model 1 and further included adiposity (BMI, WC and BF%) or walking pace. The covariates were excluded from the model if they were the exposure. The reference value was brisk walking pace, normal weight (based on BMI), low WC, or low BF% for each analysis. Correlations between BMI, WC and BF% were tested using pairwise correlation coefficients. A sensitivity analysis was conducted by adding medication (including cardiovascular medications, thiazides, steroids and statins) as a covariate to the models.

The combined associations of adiposity levels and walking pace with incident type 2 diabetes were analysed in women and men separately. All analyses were adjusted for age, deprivation index, education, smoking status, fruit and vegetable intake, red meat intake, processed meat intake, alcohol consumpation, total sedentary time, sleep duration, and multimorbidity or medication for cardiometabolic diseases. The reference value was the combination of normal BMI, low WC, or low BF% with brisk walking pace.

As the magnitude of the association between walking pace and incident type 2 diabetes was attenuated after adjusting for adiposity, a four‐way decomposition analysis^18^ was conducted to examine whether the excess risk of slow walking pace could be attributed to mediation via adiposity and/or interaction with adiposity (BMI, WC and BF%). Briefly, the excess risk of slow walking pace was split into a controlled direct effect (unrelated to adiposity), pure interaction, mediated interaction, and pure mediation. For this study, mediated interaction and pure mediation were combined and called “total mediation”. This was formally tested in a causal counterfactual framework, as described elsewhere,^19^, ^20^, ^21^ using the CMAverse package in R.^22^ The model was adjusted for age, deprivation index, education, smoking status, fruit and vegetable intake, red meat intake, processed meat intake, alcohol consumption, total sedentary time, sleep duration and multimorbidity (Model 1).

Nonlinear analysis was also conducted to investigate the associations of adiposity and walking pace with incident type 2 diabetes. Firstly, adiposity was fitted into the model as a continuous variable. Nonlinear associations were examined using penalized cubic splines fitted in Cox proportional hazard models. The penalized spline is not as sensitive to knot numbers and placements as restricted cubic splines.^23^, ^24^ We used likelihood ratio tests to test nonlinearity in exposure‐outcome relationships by comparing models with adiposity splines and models with linear adiposity terms.

Statistical analyses were performed using the statistical software stata 17 (StataCorp LP) and R v4.0.2. Significance was accepted at a *P* value < 0.05. The Anderson‐Darling test was conducted to check the normality of variables.

### 2.7 Ethics statement

The UK Biobank study was approved by the North‐West Multi‐Centre Research Ethics Committee (ref. 11/NW/0382 on June 17, 2011). All participants provided written informed consent to participate in the UK Biobank study. The study protocol is available online (http://www.ukbiobank.ac.uk/). This research has been conducted using the UK Biobank resource under application number 7155.

## 3 RESULTS

In the 2‐year landmark analyses and after excluding participants with prevalent diabetes, undiagnosed diabetes or non‐White Europeans at baseline, 194 304 participants with data available for incident type 2 diabetes, adiposity, walking pace and covariates were included in this study (Figure S1). The median (interquartile range) follow‐up period was 5.4 (4.8‐6.3) years. Over the follow‐up, 4564 participants (2.3%) developed type 2 diabetes, including 1916 women (42.0%) and 2648 men (58.0%).

There was a high correlation between BMI and WC (r = 0.81) but a moderate to low correlation between BMI and BF% (r = 0.57) as well as WC and BF% (r = 0.24).

Table 1 shows the cohort characteristics of the men and women included in this study. In summary, the mean ± SD age was 56.5 ± 8.0 years, with 56.4% women. A higher proportion of the participants reported having college or university degrees and being never‐smokers. Men, compared to women, reported a higher intake of red and processed meat but lower alcohol consumption and fruit and vegetable intake. A higher prevalence of overweight was observed for men compared to women (Table 1).

**TABLE 1:** Characteristics of the study cohort, stratified by sex

Characteristics	Overall n = 194 304	Women n = 108 631 (55.9%)	Men n = 85 673 (44.1%)
Age, years (mean ± SD)	56.5 ± 8.0	56.4 ± 7.9	56.7 ± 8.1
Townsend deprivation index, n (%)			
Low deprivation	68 023 (35.0)	37 708 (34.7)	30 315 (35.4)
Middle deprivation	67 521 (34.8)	38 073 (35.1)	29 448 (34.4)
High deprivation	58 760 (30.2)	32 850 (30.2)	25 910 (30.2)
Education, n (%)			
College or university degree	73 287 (45.8)	40 073 (45.0)	33 214 (46.9)
A levels/AS levels or equivalent	21 626 (13.5)	12 759 (14.3)	8867 (12.5)
O levels/GCSEs or equivalent	41 902 (26.2)	25 682 (28.8)	16 220 (22.9)
CSEs or equivalent/NVQ or HND or HNC	23 052 (14.4)	10 534 (11.8)	12 518 (17.7)
Lifestyle			
Smoking status, n (%)			
Never	68 023 (35.0)	37 708 (34.7)	30 315 (35.4)
Previous	67 521 (34.8)	38 073 (35.1)	29 448 (34.4)
Current	58 760 (30.2)	32 850 (30.2)	25 910 (30.2)
Sleep duration, h/d (mean ± SD)	7.2 ± 1.1	7.2 ± 1.1	7.1 ± 1.0
Red meat intake, portions/week (mean ± SD)	2.1 ± 1.4	2.0 ± 1.3	2.3 ± 1.5
Process meat intake, portions/week (mean ± SD)	1.9 ± 1.1	1.6 ± 1.0	2.2 ± 1.0
Alcohol intake (mean ± SD)	2.9 ± 1.5	3.1 ± 1.5	2.6 ± 1.4
Fruit and vegetable intake, g/d (mean ± SD)	324.4 ± 185.2	346.3 ± 181.8	296.6 ± 185.8
Total sedentary time, h/d (mean ± SD)	5.0 ± 2.2	4.7 ± 2.0	5.4 ± 2.4
Walking pace, n (%)			
Brisk	80 142 (41.2)	43 901 (40.4)	36 241 (42.3)
Average	101 408 (52.2)	57 181 (52.6)	44 227 (51.6)
Slow	12 754 (6.6)	7549 (7.0)	5205 (6.1)
Adiposity			
Waist circumference, cm (mean ± SD)	89.5 ± 12.9	84.1 ± 11.9	96.3 ± 10.7
Body fat percentage (mean ± SD)	31.6 ± 8.5	36.7 ± 6.7	25.1 ± 5.6
BMI, kg/m^2^ (mean ± SD)	27.3 ± 4.5	27.0 ± 4.9	27.7 ± 4.0
BMI category, n (%)			
Normal weight	65 092 (33.5)	43 163 (39.7)	21 929 (25.6)
Overweight	84 812 (43.7)	41 241 (38.0)	43 571 (50.9)
Obesity	44 400 (22.9)	24 227 (22.3)	20 173 (23.6)
Multimorbidity, n (%)			
None	69 889 (36.0)	37 889 (34.9)	32 000 (37.4)
1	66 092 (34.0)	36 436 (33.5)	29 656 (34.6)
2	35 854 (18.5)	20 425 (18.8)	15 429 (18.0)
3	14 846 (7.6)	8902 (8.2)	5944 (6.9)
4	5200 (2.7)	3315 (3.1)	1885 (2.2)
≥5	2423 (1.2)	1664 (1.5)	759 (0.9)

*Note*: Data are presented as mean and SD for continuous variables and frequency and % for categorical variables.

Abbreviations: A levels/AS levels, Advanced/Advanced Subsidiary Levels; BMI, body mass index; CSEs, Certificate of Secondary Educaions; GCSE, General Certificate of Secondary Education; HNC, Higher National Certificate; HND, Higher National Diploma; NVQ, National Vocational Qualification; O levels, ordinary levels.

The associations between walking pace and incident type 2 diabetes for women and men are presented in Table 2. Compared to a brisk walking pace, women who reported either average or slow walking paces had a 75% (HR 1.75 [95% CI 1.55; 1.96]) or 2.5 times (HR 2.52 [95% CI 2.15; 2.94]) higher risk of type 2 diabetes, respectively, after adjusting for sociodemographic and lifestyle confounding factors (Model 1). When the analysis was further adjusted for adiposity, the risk of type 2 diabetes was attenuated for slow walking pace, therefore, mediation analyses were conducted to test if adiposity mediated the associaion. In men, compared to a brisk walking pace, after adjusting for sociodemographic and lifestyle factors (Model 1), individuals who reported average and slow walking paces had a 60% (HR 1.60 [95% CI 1.45; 1.75]) and 2.1 times (HR 2.05 [95% CI 1.78; 2.35)] higher risk of type 2 diabetes, respectively. The magnitude of risk of type 2 diabetes was attenuated but still significant after further adjusting for adiposity (Model 2) to a 21% (HR 1.21 [95% CI 1.10; 1.33]) and 18% (HR 1.18 [95% CI 1.02; 1.36]) higher risk, respectively. The magnitudes and directions of the associations between walking pace, adiposity and type 2 diabetes were similar when medication was added to the most adjusted model (Model 2; Tables S1).

**TABLE 2:** Association of walking pace and adiposity with incident type 2 diabetes

	Women				Men			
	Model 1		Model 2		Model 1		Model 2	
	HR (95% CI)	*P* value	HR (95% CI)	*P* value	HR (95% CI)	*P* value	HR (95% CI)	*P* value
Walking pace								
Brisk	1.00 (ref.)		1.00 (ref.)		1.00 (ref.)		1.00 (ref.)	
Average	1.75 (1.55; 1.96)	<0.0001	1.21 (1.08; 1.37)	0.002	1.60 (1.45; 1.75)	<0.0001	1.21 (1.10; 1.33)	<0.0001
Slow	2.52 (2.15; 2.94)	<0.0001	1.10 (0.94; 1.30)	0.242	2.05 (1.78; 2.35)	<0.0001	1.18 (1.02; 1.36)	0.024
Trend	1.59 (1.48; 1.72)	<0.0001	1.06 (0.98; 1.15)	0.160	1.46 (1.37; 1.56)	<0.0001	1.11 (1.04; 1.19)	0.002
BMI								
Normal weight	1.00 (ref.)		1.00 (ref.)		1.00 (ref.)		1.00 (ref.)	
Overweight	3.43 (2.86; 4.12)	<0.0001	3.36 (2.80; 4.02)	<0.0001	3.54 (2.95; 4.24)	<0.0001	3.48 (2.91; 4.17)	<0.0001
Obesity	10.05 (8.44; 11.97)	<0.0001	9.44 (7.91; 11.27)	<0.0001	10.12 (8.47; 12.11)	<0.0001	9.62 (8.04; 11.52)	<0.0001
Trend	3.07 (2.86; 3.30)	<0.0001	2.97 (2.76; 3.20)	<0.0001	3.01 (2.82; 3.21)	<0.0001	2.92 (2.73; 3.11)	<0.0001
WC								
Low WC	1.00 (ref.)		1.00 (ref.)		1.00 (ref.)		1.00 (ref.)	
Middle WC	3.48 (2.80; 4.33)	<0.0001	3.41 (2.74; 4.24)	<0.0001	2.46 (2.14; 2.82)	<0.0001	2.42 (2.1; 2.78)	<0.0001
Hight WC	12.16 (9.91; 14.91)	<0.0001	11.44 (9.32; 14.06)	<0.0001	6.53 (5.74; 7.42)	<0.0001	6.24 (5.48; 7.10)	<0.0001
Trend	3.49 (3.22; 3.78)	<0.0001	3.37 (3.11; 3.66)	<0.0001	2.59 (2.44; 2.74)	<0.0001	2.52 (2.38; 2.67)	<0.0001
BF%								
Low BF%	1.00 (ref.)		1.00 (ref.)		1.00 (ref.)		1.00 (ref.)	
Middle BF%	3.14 (2.56; 3.84)	<0.0001	3.05 (2.49; 3.74)	<0.0001	2.77 (2.39; 3.21)	<0.0001	2.72 (2.34; 3.16)	<0.0001
Hight BF%	7.91 (6.53; 9.59)	<0.0001	7.30 (6.01; 8.87)	<0.0001	6.47 (5.62; 7.45)	<0.0001	6.15 (5.34; 7.09)	<0.0001
Trend	2.69 (2.49; 2.90)	<0.0001	2.57 (2.38; 2.78)	<0.0001	2.47 (2.33; 2.62)	<0.0001	2.40 (2.26; 2.55)	<0.0001

*Note*: Data are presented as HR and 95% CI. Model 1 was adjusted for age, deprivation index, education, smoking status, fruit and vegetable, red meat and processed meat intake, alcohol consumption, total sedentary time, sleep duration and multimorbidity. Model 2 was adjusted as for Model 1 but further included walking pace when BMI, WC and BF% were the exposures and vice versa. All analyses were conducted using 2‐year landmark analyses, excluding participants with type 1, type 2 diabetes, unknown diabetes and non‐White ethnic background.

Abbreviations: BF%, body fat percentage; BMI, body mass index; CI, confidence interval; HR, hazard ratio; WC, waist circumference.

Adiposity was positively associated with a higher risk of type 2 diabetes regardless of the adiposity marker used. Compared to normal weight as determined by BMI, both women and men who were living with overweight or obesity had a three or nine times higher risk of type 2 diabetes. For WC, women and men who were in the high WC category had an 11.4 times (HR 11.44 [95% CI 9.32; 14.06]) and 6.2 times (HR 6.24 [95% CI 5.48; 7.10]) higher risk of type 2 diabetes, respectively, compared to the lowest category. For BF%, women and men who were in the high BF% category had a 7.3 times (HR 7.30 [95% CI: 6.01; 8.87]) and 6.2 times (HR 6.15 [95% CI: 5.34; 7.09]) higher risk of type 2 diabetes, respectively, compared to the low category, as shown in Table 2.

**FIGURE 1:**
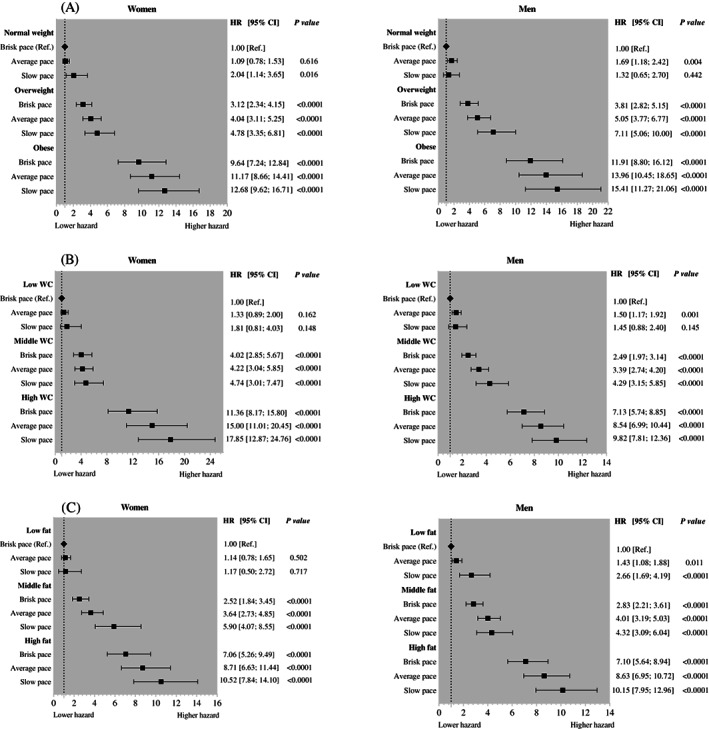
Combined association between adiposity and walking pace and incident type 2 diabetes. Data are presented as hazard ratio (HR) and 95% confidence interval (CI). Analyses were adjusted for age, deprivation index, education, smoking status, fruit and vegetable intake, red meat intake, processed meat intake, alcohol consumption, total sedentary time, sleep duration and multimorbidity. All analyses were conducted using 2‐year landmark analyses and excluding participants with type 1, type 2 diabetes, unknown diabetes and non‐White ethnic background. A, Combined association between body mass index and walking pace and incident type 2 diabetes. B, Combined association between waist circumference (WC) and walking pace and incident type 2 diabetes. C, Combined association between body fat percentage and walking pace and incident type 2 diabetes

Figure 1 shows the combined association between adiposity, walking pace, and incident type 2 diabetes. The risk of type 2 diabetes was higher when walking pace was slow. Among women, participants with obesity, central obesity or had high BF% and also reported a slow walking pace had a 12.7 times (HR 12.68 [95% CI 9.62; 16.71]), 17.9 times (HR 17.85 [95% CI 12.87; 24.78]) or 10.5 times (HR: 10.52 [95% CI 7.84; 14.10]) higher risk of developing type 2 diabetes compared to those with normal BMI, low WC or low BF% with brisk walking pace, respectively. Among men, participants with obesity, central obesity or had high BF% and also reported a slow pace had a 15.4 times (HR 15.41 [95% CI 11.27; 21.06]), 9.8 times (HR 9.82 [95% CI 7.81; 12.36]) or 10.2 times (HR 10.15 [95% CI 7.95; 12.96]) higher risk of developing type 2 diabetes compared to those with normal BMI, low WC or low BF% with brisk walking pace, respectively. Similar results were found when medication was added to the model as a covariate (Figure S2).

General and central obesity were partial mediators for the association in women, accounting for 60.1% and 64.4%, respectively. There was evidence of an additive interaction of WC and body fat with walking pace in women; however, only body fat showed an interaction with walking pace in men (Table 3). The mediation analysis showed that BF% (Table 3) mediated 44.9% (for women) and 35.5% (for men) of the excess risk due to slow walking. Being a slow walker and having a high BF% would have a synergistic deleterious association, explaining another 47.9% and 26.5% of the excess risk for women and men, respectively. The total effect, total natural indirect effect and pure natural direct effect are shown in Table S2.

**TABLE 3:** Four‐way decomposition analysis of walking pace and adiposity with type 2 diabetes

Proportion of excess risk of slow walking due to	General obesity	Central obesity	High body fat percentage
Women			
Additive interaction (%)	1.6 (−6.3; 6.8)	17.8 (9.4; 23.8)*	47.9 (34.2; 58.0)*
Total mediation (%)	60.1 (51.8; 69.7)*	64.4 (57.2; 73.4)*	44.9 (38.2; 50.8)*
Men			
Additive interaction (%)	2.7 (−8.3; 10.3)	6.7 (−5.7; 15.2)	25.6 (12.3; 36.7)*
Total mediation (%)	44.9 (36.9; 52.3)*	44.7 (37.2; 52.9)*	35.5 (23; 42.7)*

*Note*: Numbers presented are percentages with 95% confidence interval. The analysis was adjusted for age, deprivation index, education, smoking status, fruit and vegetable intake, red meat intake, processed meat intake, alcohol consumption, total sedentary time, sleep duration and multimorbidity. All analyses were conducted using 2‐year landmark analyses, excluding participants with type 1, type 2 diabetes, unknown diabetes and non‐White ethnic background. Obesity was defined as BMI ≥30 kg/m^2^; central obesity was defined as WC ≥88 cm for women and ≥102 cm for men; BF% was defined as >35% for women and >25% for men.

Abbreviations: BF%, body fat percentage; BMI, body mass index; WC, waist circumference.

* *P* < 0.0001.

**FIGURE 2:**
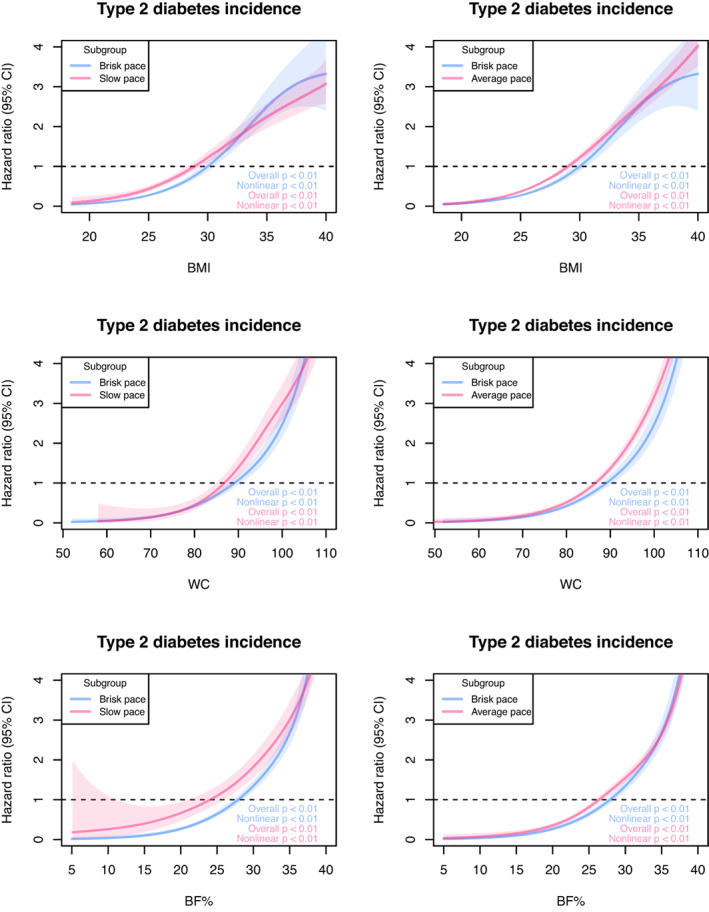
Penalized cubic splines for the association between walking pace, adiposity and incident type 2 diabetes. Data are presented as hazard ratios and their 95% confidence interval (CI). Analyses were adjusted for age, deprivation index, education, smoking status, fruit and vegetable intake, red meat intake, processed meat intake, alcohol consumption, total sedentary time, sleep duration and multimorbidity. All analyses were conducted using 2‐year landmark analyses and excluding participants with type 1, type 2 diabetes, unknown diabetes and non‐white ethnic background. BF%, body fat percentage; BMI, body mass index; WC, waist circumference

Positive nonlinear associations were found between adiposity, walking pace and incident type 2 diabetes (Figure 2). Compared to brisk walking pace, the spline for slow walking pace showed that type 2 diabetes risk increased with increasing BMI up to 33 kg/m^2^, after which the lower risk appeared. For WC, the risk of incident type 2 diabetes for slow walking pace increased sharply with greater WC up to 103 cm, then converged. The spline for slow walking pace showed that the risk of incident type 2 diabetes seemed to increase sharply with increasing BF% up to 36%, then converge.

## 4 DISCUSSION

Compared to brisk walking pace, slow walking pace was associated with a higher risk of type 2 diabetes, independent of sociodemographic and lifestyle factors among White‐European people. This association was largely explained by adiposity in women but not in men. The risk of type 2 diabetes was doubled when slow walking pace was combined with obesity, high WC or BF%. These findings suggest that both walking pace and adiposity are independent risk factors for type 2 diabetes and that they have to be considered simultaneously to achieve the maximum public health benefits. Importantly, as adiposity could be a mediator between walking pace and type 2 diabetes, promoting brisk walking for weight management could have synergistic benefits for type 2 diabetes prevention. That noted, it should be clear that excess adiposity, however measured, was the dominant risk factor for type 2 diabetes so that brisk walking on its own is unlikely to be sufficient to prevent type 2 diabetes if there are other reasons, for example, poor diet, that lead to weight excess.

Our study is consistent with previous evidence on walking pace and the risk of type 2 diabetes. A study from the United States, conducted in 70 102 women aged 40 to 60 years, found that after adjusting for age, time period, cigarette smoking, menopausal status, parental history of diabetes, alcohol consumption, history of hypertension, history of high cholesterol level and time spent walking per week, normal and brisk walking pace were associated with a 28% (relative risk [RR] 0.72 [95% CI 0.62; 0.85]) and 59% (RR 0.41 [95% CI 0.33; 0.52]) lower risk of type 2 diabetes, respectively, compared to an easy walking pace.^25^ However, after adjusting for BMI, the association for brisk walkers was slightly attenuated (RR 0.59 [95% CI 0.47; 0.73]) but remained significant.^25^ The Health Professional's Follow‐up Study, conducted in the United States in 37 918 men aged 40 to 75 years, reported that, compared to slow walkers, those who reported a normal, brisk or very brisk walking pace had a 32%, 54% and 61% lower risk of type 2 diabetes independent of BMI.^26^

Current evidence mainly focuses on stratified analysis or including adiposity as a covariate in the statistical analysis.^11^ However, some studies have reported the joint associations between total physical activity and BMI with type 2 diabetes risk.^7^, ^8^ A more recent meta‐analysis reported a seven times higher risk of type 2 diabetes in people with obesity and who had low physical activity compared to those with normal weight and high physical activity.^8^ Likewise, our findings showed that the combination of obesity and slow walking pace was associated with an approximately 10 times higher risk of type 2 diabetes, compared to normal weight and brisk walking pace.

Interestingly, four‐way decomposition analysis in our findings showed the excess risk due to slow walking was mainly mediated through general and central obesity but had an additive interaction with body fat proportion. This indicates that people with high body fat are more vulnerable to slow walking and that increasing their walking speed during physical activity, where feasible, could potentially be effective as it could reduce body fat. Randomized controlled trials are warranted to test these hypotheses.

Our study is an observational study, so we cannot interpret causality directly. However, the association is biologically plausible. Walking, as an aerobic exercise, can improve insulin action in the muscles and liver by increasing glucose uptake acutely and during prolonged activity.^27^ Furthermore, a previous Mendelian randomization study has suggested walking pace to be a potential causal factor for various health outcomes.^28^ In contrast, it is well known that obesity is the strongest risk factor for type 2 diabetes.^29^ Adiposity composition and sex hormones might explain why some of the findings differed by sex.^30^, ^31^

The strengths of the present study include its large number of participants, which provided a sufficient sample size to undertake the analysis. We included multiple confounding factors, which enabled the research question to be sufficiently adjusted and investigated. A 2‐year landmark analysis likely reduces the risk of reverse causation impacting the results and is a strength of this study. Walking pace is a low cost equipment and is easy to administer and would therefore be relatively simple to assess in clinical practice. Although our study used self‐reported usual walking pace, there is evidence that self‐reported walking speed is a valuable marker of walking speed measurement and could be a marker of physical performance when walking speed is not possible to measure directly in sarcopenia and frailty.^32^ Self‐reporting walking pace does not consider the time spent walking, which could modify the association between walking pace and obesity in type 2 diabetes. However, a previous study has shown that the association between walking pace and diabetes risk is independent of time spent walking.^33^ It is well known that BMI has limitations when used to assess obesity,^3^ but that WC is a better predictor for evaluating the risk of type 2 diabetes.^34^ Our study provided evidence that adiposity and fat distribution, BMI, WC or BF%, combined with a measure of physical activity such as walking pace, showed a strong relationship to type 2 diabetes risk.

The present study also has several limitations. The UK Biobank is not representative of the general population of the United Kingdom with regard to sociodemographic, physical, lifestyle and health‐related characteristics. Although the UK Biobank data have been showed to have healthy volunteer selection bias, exposure‐disease risk estimates can nevertheless be generalized to the broader population.^35^ In addition, walking pace was self‐reported, however, previous studies have provided robust evidence of the predictive ability of self‐reported walking pace above and beyond traditional risk factors.^9^, ^10^ We were also unable to include all ethnicities as there was insufficient statistical power to detect differences according to ethnicity. Although standardized protocols were implemented across the assessment centres, the data do not allow us to quantify the accuracy and repeatability of the methods used. Reverse causation due to the link between existing multimorbidity and walking pace cannot be ignored; however, when the analyses were adjusted for multimorbidity or medication, the associations remained similar. Although these sensitivity analyses cannot fully remove the effect of reverse causation, they provide evidence that reverse causation does not fully explain the associations observed.

To conclude, walking pace was inversely associated with the risk of type 2 diabetes for both men and women, independent of sociodemographic characteristics, lifestyle and adiposity. Slow walking pace, combined with obesity, high WC and BF%, doubled the risks of developing type 2 diabetes. Promoting brisk walking as a weight management measure might be an effective type 2 diabetes prevention strategy, given its synergistic effect.

## AUTHOR CONTRIBUTIONS

Jirapitcha Boonpor, Stuart R. Gray, Frederick K. Ho and Carlos Celis‐Morales contributed to the study conception and design, advised on all statistical aspects, and interpreted the data. Jirapitcha Boonpor, Solange Parra‐Soto, Atefeh Talebi, Frederick K. Ho and Carlos Celis‐Morales performed the statistical analyses. Jirapitcha Boonpor, Stuart R. Gray, Frederick K. Ho and Carlos Celis‐Morales drafted the manuscript. All authors reviewed the manuscript and approved the final version to be published. Stuart R. Gray, Frederick K. Ho and Carlos Celis‐Morales are the guarantors of this work and, as such, had full access to all the data in the study and take responsibility for the integrity of the data and the accuracy of the data analysis.

## FUNDING INFORMATION

The UK Biobank was established by the Wellcome Trust, Medical Research Council, Department of Health, Scottish Government, and the Northwest Regional Development Agency. It has also received funding from the Welsh Assembly Government and the British Heart Foundation. Jirapitcha Boonpor receives financial support from the Royal Thai Government Scholarship for her PhD. Solange Parra‐Soto receives financial support from the Chilean Government PhD scholarship program for their PhD. The funders have no role in study design, in the collection, analysis or interpretation of data, in the report's writing, or in the decision to submit the paper for publication.

## CONFLICT OF INTEREST STATEMENT

The authors declare no conflicts of interest.

### PEER REVIEW

The peer review history for this article is available at https://www.webofscience.com/api/gateway/wos/peer-review/10.1111/dom.15053.

## Supporting information


**Data S1.** Supporting Information.

## Data Availability

Data can be requested from the UK Biobank (https://www.ukbiobank.ac.uk/).

## References

[1] International diabetes federation . IDF Diabetes atlas ninth edition. 2019 www.diabetesatlas.org

[2] Saeedi P , Petersohn I , Salpea P , et al. Global and regional diabetes prevalence estimates for 2019 and projections for 2030 and 2045: results from the international diabetes federation diabetes atlas, 9(th) edition. Diabetes Res Clin Pract. 2019;157:107843. 31518657 10.1016/j.diabres.2019.107843

[3] Piché M‐E , Tchernof A , Després J‐P . Obesity phenotypes, diabetes, and cardiovascular diseases. Circ Res. 2020;126(11):1477‐1500. 32437302 10.1161/CIRCRESAHA.120.316101

[4] Verma S , Hussain ME . Obesity and diabetes: an update. Diabetes Metab Syndr. 2017;11(1):73‐79. 27353549 10.1016/j.dsx.2016.06.017

[5] Chan RS , Woo J . Prevention of overweight and obesity: how effective is the current public health approach. Int J Environ Res Public Health. 2010;7(3):765‐783. 20617002 10.3390/ijerph7030765 PMC2872299

[6] Hu G , Lindström J , Valle TT , et al. Physical activity, body mass index, and risk of type 2 diabetes in patients with Normal or impaired glucose regulation. Arch Intern Med. 2004;164(8):892‐896. 15111376 10.1001/archinte.164.8.892

[7] Weinstein AR , Sesso HD , Lee IM , et al. Relationship of physical activity vs body mass index with type 2 diabetes in women. JAMA. 2004;292(10):1188‐1194. 15353531 10.1001/jama.292.10.1188

[8] Cloostermans L , Wendel‐Vos W , Doornbos G , et al. Independent and combined effects of physical activity and body mass index on the development of type 2 diabetes – a meta‐analysis of 9 prospective cohort studies. Int J Behav Nutr Phys Act. 2015;12(1):147. 26619831 10.1186/s12966-015-0304-3 PMC4666059

[9] Ganna A , Ingelsson E . 5 year mortality predictors in 498 103 UK Biobank participants: a prospective population‐based study. Lancet. 2015;386(9993):533‐540. 26049253 10.1016/S0140-6736(15)60175-1

[10] Welsh CE , Celis‐Morales CA , Ho FK , et al. Grip strength and walking pace and cardiovascular disease risk prediction in 406,834 UK Biobank participants. Mayo Clin Proc. 2020;95(5):879‐888. 32299669 10.1016/j.mayocp.2019.12.032

[11] Iwasaki M , Kudo A , Asahi K , et al. Fast walking is a preventive factor against new‐onset diabetes mellitus in a large cohort from a Japanese general population. Sci Rep. 2021;11(1):716. 33436978 10.1038/s41598-020-80572-y PMC7804125

[12] Collins R . What makes UK Biobank special? Lancet. 2012;379(9822):1173‐1174. 22463865 10.1016/S0140-6736(12)60404-8

[13] Sudlow C , Gallacher J , Allen N , et al. UK biobank: an open access resource for identifying the causes of a wide range of complex diseases of middle and old age. PLoS Med. 2015;12(3):e1001779. 25826379 10.1371/journal.pmed.1001779 PMC4380465

[14] UK Biobank . UK Biobank: protocol for a large‐scale prospective epidemiological resource. 2007; http://www.ukbiobank.ac.uk/wp-content/uploads/2011/11/UK-Biobank-Protocol.pdf. Accessed July, 2020.

[15] Hardman AE , Stensel DJ . Obesity and energy balance. Physical Activity and Health: the Evidence Explained. Vol 1. London and New York: Routledge; 2003:114‐130.

[16] Celis‐Morales CA , Gray S , Petermann F , et al. Walking pace is associated with lower risk of all‐cause and cause‐specific mortality. Med Sci Sports Exerc. 2019;51(3):472‐480. 30303933 10.1249/MSS.0000000000001795

[17] Townsend NP , Phillimore P , Beattie A . Health and Deprivation: Inequality and the North. London: Croom Helm Ltd; 1988.

[18] VanderWeele TJ . A unification of mediation and interaction: a 4‐way decomposition. Epidemiology. 2014;25(5):749‐761. 25000145 10.1097/EDE.0000000000000121 PMC4220271

[19] Tingley D , Yamamoto T , Hirose K , Keele L , Imai K . Mediation: R package for causal mediation analysis. J Stat Softw. 2014;59(5):1‐38.26917999

[20] VanderWeele TJ , Tchetgen Tchetgen EJ . Mediation analysis with time varying exposures and mediators. J R Stat Soc Ser B Stat Methodol. 2017;79(3):917‐938. 10.1111/rssb.12194 PMC5560424 28824285

[21] Boonpor J , Petermann‐Rocha F , Parra‐Soto S , et al. Types of diet, obesity, and incident type 2 diabetes: findings from the UK Biobank prospective cohort study. Diabetes Obes Metab. 2022;24(7):1351‐1359. 35373896 10.1111/dom.14711 PMC9325356

[22] Shi B , Choirat C , Coull BA , VanderWeele TJ , Valeri L . CMAverse: a suite of functions for reproducible causal mediation analyses. Epidemiology. 2021;32(5):e20‐e22. 34028370 10.1097/EDE.0000000000001378

[23] Govindarajulu US , Malloy EJ , Ganguli B , Spiegelman D , Eisen EA . The comparison of alternative smoothing methods for fitting non‐linear exposure‐response relationships with cox models in a simulation study. Int J Biostat. 2009;5(1):Article 2. 20231865 10.2202/1557-4679.1104 PMC2827890

[24] Ho, FK , Cole TJ. Non‐linear predictor outcome associations. BMJ Medicine, 2023;2(1):e000396. doi:10.1136/bmjmed-2022-000396 PMC9951370 36936259

[25] Hu FB , Sigal RJ , Rich‐Edwards JW , et al. Walking compared with vigorous physical activity and risk of type 2 diabetes in women a prospective study. JAMA. 1999;282(15):1433‐1439. 10535433 10.1001/jama.282.15.1433

[26] Hu FB , Leitzmann MF , Stampfer MJ , Colditz GA , Willett WC , Rimm EB . Physical activity and television watching in relation to risk for type 2 diabetes mellitus in men. Arch Intern Med. 2001;161(12):1542‐1548. 11427103 10.1001/archinte.161.12.1542

[27] Colberg SR , Sigal RJ , Yardley JE , et al. Physical activity/exercise and diabetes: a position Statement of the American Diabetes Association. Diabetes Care. 2016;39(11):2065‐2079. 27926890 10.2337/dc16-1728 PMC6908414

[28] Timmins IR , Zaccardi F , Nelson CP , Franks PW , Yates T , Dudbridge F . Genome‐wide association study of self‐reported walking pace suggests beneficial effects of brisk walking on health and survival. Commun Biol. 2020;3(1):634. 33128006 10.1038/s42003-020-01357-7 PMC7599247

[29] Carbone S , Del Buono MG , Ozemek C , Lavie CJ . Obesity, risk of diabetes and role of physical activity, exercise training and cardiorespiratory fitness. Prog Cardiovasc Dis. 2019;62(4):327‐333. 31442513 10.1016/j.pcad.2019.08.004

[30] Mauvais‐Jarvis F . Epidemiology of gender differences in diabetes and obesity. In: Mauvais‐Jarvis F , ed. Sex and Gender Factors Affecting Metabolic Homeostasis, Diabetes and Obesity. Cham: Springer International Publishing; 2017:3‐8. 10.1007/978-3-319-70178-3_1 29224087

[31] Sattar N . Type 2 diabetes‐related sex differences in cardiovascular risk: reasons, ramifications, and clinical realities. Eur Heart J. 2020;41(13):1354‐1356. 31860071 10.1093/eurheartj/ehz914

[32] Syddall HE , Westbury LD , Cooper C , Sayer AA . Self‐reported walking speed: a useful marker of physical performance among community‐dwelling older people? J Am Med Dir Assoc. 2015;16(4):323‐328. 25523286 10.1016/j.jamda.2014.11.004 PMC6600869

[33] Boonpor J , Ho FK , Gray SR , Celis‐Morales CA . Association of Self‐reported Walking Pace with Type 2 diabetes incidence in the UK Biobank prospective cohort study. Mayo Clin Proc. 2022;97(9):1631‐1640. 36058577 10.1016/j.mayocp.2022.02.028

[34] Schulze MB , Thorand B , Fritsche A , et al. Body adiposity index, body fat content and incidence of type 2 diabetes. Diabetologia. 2012;55(6):1660‐1667. 22349074 10.1007/s00125-012-2499-z

[35] Fry A , Littlejohns TJ , Sudlow C , et al. Comparison of sociodemographic and health‐related characteristics of UK Biobank participants with those of the general population. Am J Epidemiol. 2017;186(9):1026‐1034. 28641372 10.1093/aje/kwx246 PMC5860371

