# Supplementary material for: Association between walking pace and incident type 2 diabetes by adiposity level: A prospective cohort study from the UK Biobank
Source: Diabetes Obes Metab. 2023 Apr 3;25(7):1900–10. doi: 10.1111/dom.15053 (PMC10947435; doi:10.1111/dom.15053)
Supplement: Supplementary file 1 — Data S1. Supporting Information. [file DOM-25-1900-s001.docx]

**SUPPLEMENTARY MATERIALS**

**Supplementary Table S1**. Sensitivity analysis of the association of walking pace and adiposity with incident type 2 diabetes. Models adjusted for medication.

|  | **Women** | | | | | **Men** | | | | |
| --- | --- | --- | --- | --- | --- | --- | --- | --- | --- | --- |
|  | Model 1 | | Model 2 | | Model 1 | | | Model 2 | |  |
|  | HR (95%CI) | p-value | HR (95%CI) | p-value | HR (95%CI) | | p-value | HR (95%CI) | p-value |  |
| **Walking pace** |  |  |  |  |  | |  |  |  |  |
| Brisk | 1.00 (Ref.) |  | 1.00 (Ref.) |  | 1.00 (Ref.) | |  | 1.00 (Ref.) |  |  |
| Average | 1.76 [1.57; 1.98] | <0.0001 | 1.21 [1.08; 1.37] | 0.002 | 1.59 [1.45; 1.74] | | <0.0001 | 1.21 [1.10; 1.33] | <0.0001 |  |
| Slow | 2.82 [2.42; 3.29] | <0.0001 | 1.17 [1.00; 1.38] | 0.055 | 2.26 [1.97; 2.59] | | <0.0001 | 1.27 [1.11; 1.46] | 0.001 |  |
| Trend | 1.68 [1.56; 1.82] | <0.0001 | 1.09 [1.00; 2.57] | 0.037 | 1.52 [1.42; 1.62] | | <0.0001 | 1.14 [1.07; 1.22] | <0.0001 |  |
| **BMI** |  |  |  |  |  | |  |  |  |  |
| Normal weight | 1.00 (Ref.) |  | 1.00 (Ref.) |  | 1.00 (Ref.) | |  | 1.00 (Ref.) |  |  |
| Overweight | 3.42 [2.86; 4.10] | <0.0001 | 3.32 [2.77; 3.99] | <0.0001 | 3.49 [2.91; 4.18] | | <0.0001 | 3.42 [2.86; 4.10] | <0.0001 |  |
| Obesity | 10.15 [8.53; 12.09] | <0.0001 | 9.35 [7.83; 11.16] | <0.0001 | 9.95 [8.32; 11.9] | | <0.0001 | 9.35 [7.81; 11.19] | <0.0001 |  |
| Trend | 3.10 [2.88; 3.33] | <0.0001 | 2.96 [2.75; 3.19] | <0.0001 | 2.99 [2.80; 3.19] | | <0.0001 | 2.88 [2.70; 3.07] | <0.0001 |  |
| **WC** |  |  |  |  |  | |  |  |  |  |
| Low WC | 1.00 (Ref.) |  | 1.00 (Ref.) |  | 1.00 (Ref.) | |  | 1.00 (Ref.) |  |  |
| Middle WC | 3.50 [2.81; 4.35] | <0.0001 | 3.41 [2.74; 4.24] | <0.0001 | 2.45 [2.13; 2.81] | | <0.0001 | 2.40 [2.09; 2.76] | <0.0001 |  |
| Hight WC | 12.33 [10.06; 15.12] | <0.0001 | 11.41 [9.29; 14.02] | <0.0001 | 6.51 [5.72; 7.40] | | <0.0001 | 6.15 [5.41; 7.00] | <0.0001 |  |
| Trend | 3.52 [3.24; 3.81] | <0.0001 | 3.37 [3.10; 3.65] | <0.0001 | 2.58 [2.44; 2.74] | | <0.0001 | 2.51 [2.36; 2.66] | <0.0001 |  |
| **BF%** |  |  |  |  |  | |  |  |  |  |
| Low BF% | 1.00 (Ref.) |  | 1.00 (Ref.) |  | 1.00 (Ref.) | |  | 1.00 (Ref.) |  |  |
| Middle BF% | 3.16 [2.58; 3.88] | <0.0001 | 3.05 [2.49; 3.74] | <0.0001 | 2.76 [2.37; 3.20] | | <0.0001 | 2.76 [2.37; 3.20] | <0.0001 |  |
| Hight BF% | 8.09 [6.68; 9.81] | <0.0001 | 7.32 [6.03; 8.89] | <0.0001 | 6.46 [5.61; 7.44] | | <0.0001 | 6.46 [5.61; 7.44] | <0.0001 |  |
| Trend | 2.72 [2.52; 2.94] | <0.0001 | 2.58 [2.38; 2.78] | <0.0001 | 2.47 [2.33; 2.63] | | <0.0001 | 2.39 [2.25; 2.54] | <0.0001 |  |

Data are presented as hazard ratio (HR) and 95% CI. Model 1 was adjusted for age, deprivation index, education, smoking status, fruit & vegetable, red meat, processed meat, alcohol intake, total sedentary time, sleep duration and medication. Model 2 was adjusted as for model 1 but further included walking pace when body mass index, waist circumference and body fat percentage were the exposures and vice versa. All analyses were conducted using 2-year landmark analyses, excluding participants with type 1, type 2 diabetes, unknown diabetes and non-white ethnic background.

Body fat percentage: BF%; BMI: body mass index; waist circumference: WC.

**Supplementary Table S2.** Four-way decomposition analysis of walking pace and adiposity with T2D

|  | | **Mediation via**  **general obesity** | **Mediation via**  **central obesity** | **Mediation via**  **body fat percentage** |
| --- | --- | --- | --- | --- |
| **Women** | TE | 2.62 [2.22; 3.04]^*^ | 2.61 [2.22; 3.05] ^*^ | 2.58 [2.19; 3.01] ^*^ |
|  | TNIE | 1.59 [1.48; 1.71] ^*^ | 1.66 [1.56; 1.75] ^*^ | 1.38 [1.32; 1.42] ^*^ |
|  | PNDE | 1.65 [1.41; 1.94] ^*^ | 1.57 [1.35; 1.87] ^*^ | 1.87 [1.60; 2.19] ^*^ |
| **Men** | TE | 2.18 [1.91; 2.49] ^*^ | 2.16 [1.91; 2.42] ^*^ | 2.21 [1.91; 2.49] ^*^ |
|  | TNIE | 1.32 [1.25; 1.39] ^*^ | 1.32 [1.25; 1.37] ^*^ | 1.24 [1.20; 1.28] ^*^ |
|  | PNDE | 1.65 [1.45; 1.91] ^*^ | 1.64 [1.46; 1.84] ^*^ | 1.78 [1.54; 2.00] ^*^ |

^*^P<0.0001

Numbers presented are HR (95% CI).

The analysis was adjusted for age, deprivation index, education, smoking status, fruit & vegetable, red meat, processed meat, alcohol intake, total sedentary time, sleep duration and multimorbidity. All analyses were conducted using 2-year landmark analyses, excluding participants with type 1, type 2 diabetes, unknown diabetes and non-white ethnic background.

Obesity was defined as BMI ≥30kg/m^2^; central obesity was defined as WC >88 cm for women and >102 cm for men; body fat percentage (BF%) was defined as BF% >35% for women and >25% for men.

TE, Total Effect; TNIE, Total Natural Indirect Effect; PNDE, Pure Natural Direct Effect

**
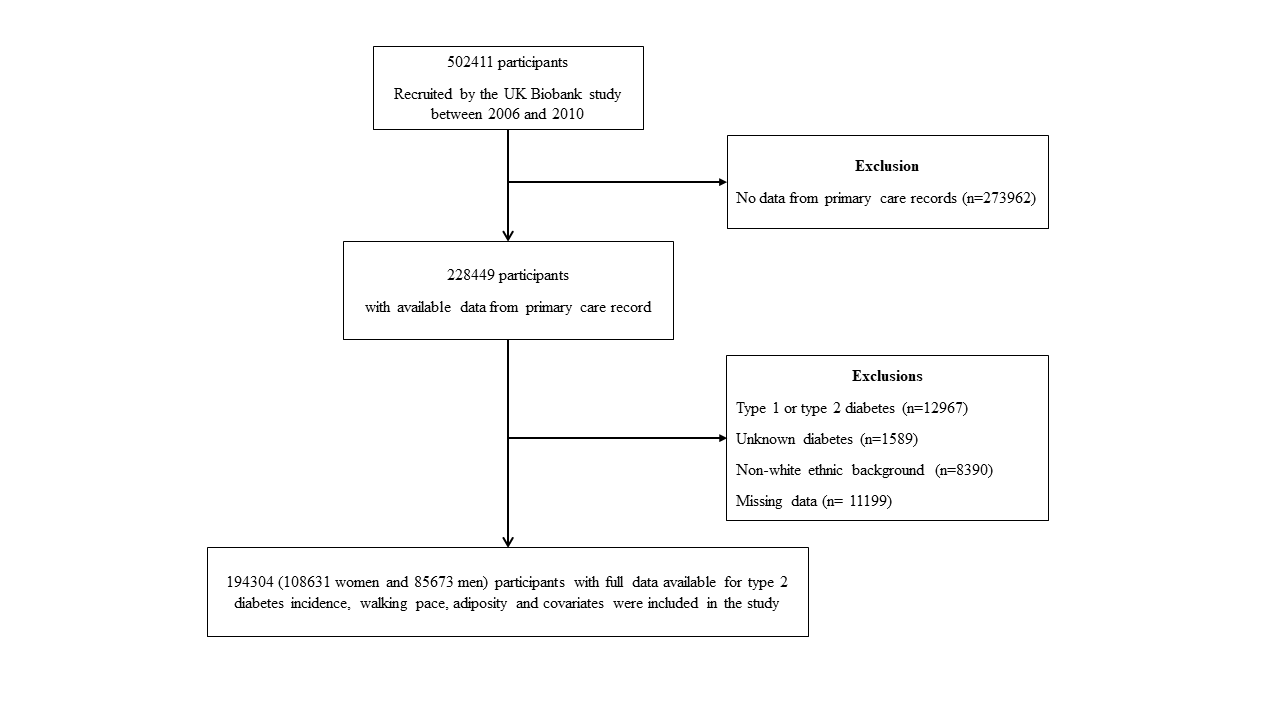
**

**Supplementary Figure S1**. Participants flowchart


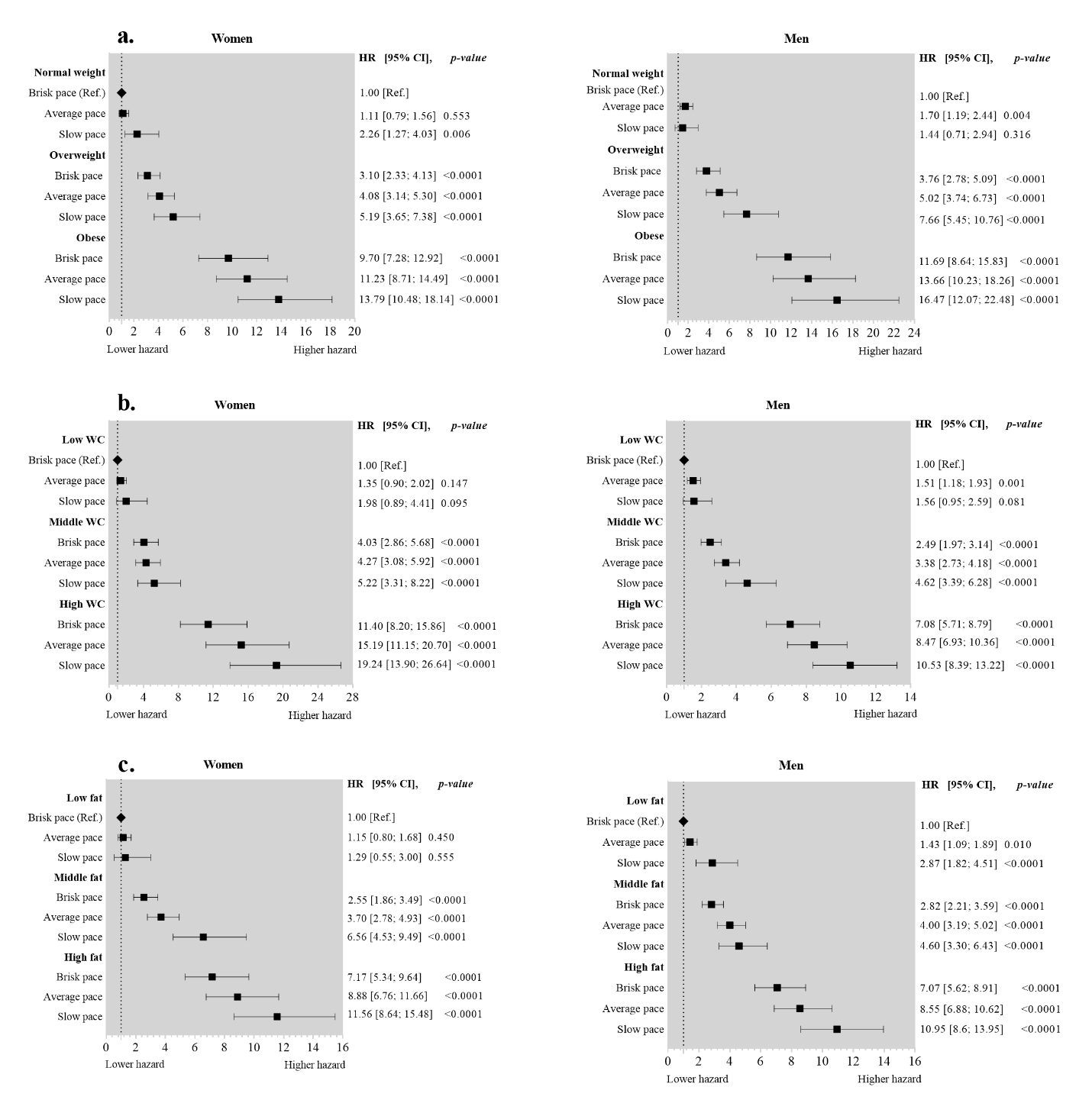


**Supplementary Figure S2**. Sensitivity analysis of the combined association between adiposity and walking pace and incident type 2 diabetes. Analysis adjusted for medication.

Data are presented as hazard ratio (HR) and 95% CI. Analyses were adjusted for age, deprivation index, education, smoking status, fruit and vegetable intake, red meat intake, processed meat intake, alcohol consumption, total sedentary time, sleep duration and medication. All analyses were conducted using 2-year landmark analyses and excluding participants with type 1, type 2 diabetes, unknown diabetes and non-white ethnic background.

Note:

a. Combined association between body mass index and walking pace and incident type 2 diabetes

b. Combined association between waist circumference and walking pace and incident type 2 diabetes

c. Combined association between body fat percentage and walking pace and incident type 2 diabetes
